# Development and optimization of thymol-loaded nanoemulsions for enhanced antimicrobial activity against methicillin-resistant *Staphylococcus aureus*

**DOI:** 10.1038/s41598-025-29481-6

**Published:** 2025-11-22

**Authors:** Lingyong Song, Weize Li, Zhizhong Hu, Yangbo Lv, Jincang Liu, Feng Cheng, Chunping Xu

**Affiliations:** 1 Technical Center of China Tobacco Guangxi Industrial Co. Ltd., Nanning, 530001 China; 2 Joint Innovation Laboratory for Natural Active Products, Henan Academy of Science, Zhengzhou, 450046 China; 3 College of Tobacco Science and Engineering, Zhengzhou University of Light Industry, Zhengzhou, 450002 China

**Keywords:** Thymol, Nanoemulsion, MRSA, Antimicrobial, Membrane disruption, Biotechnology, Drug discovery, Microbiology, Nanoscience and technology

## Abstract

The emergence of methicillin-resistant *Staphylococcus aureus* (MRSA) necessitates the development of novel antimicrobial strategies. This study developed and optimized thymol-loaded nanoemulsions (TNE) as a standardized, reproducible alternative to variable essential oil formulations using central composite design. The optimal formulation comprised 4.6% oil phase, 18.5% surfactant, and 2.5:1 surfactant-to-cosurfactant ratio, yielding nanoemulsions with particle size of 25.51 ± 0.15 nm, polydispersity index (PDI) of 0.114 ± 0.008, and zeta potential of − 2.73 ± 0.12 mV. TNE demonstrated consistent antimicrobial activity with MIC values of 15 mg/mL against MRSA and other bacterial strains. Comprehensive mechanistic studies using five complementary assays revealed that TNE exerts antimicrobial effects through multiple membrane-targeting pathways, including membrane permeabilization, depolarization, hydrophobicity alteration, and rigidity modulation. The multi-target mechanism suggests reduced likelihood of resistance development compared to conventional single-target antibiotics. These findings demonstrate that systematically optimized thymol nanoemulsions represent a promising standardized platform for combating multidrug-resistant infections, offering enhanced stability, controlled release characteristics, and reduced resistance potential.

## Introduction

Methicillin-resistant Staphylococcus aureus (MRSA) represents a major global healthcare challenge, with approximately 120,000 Staphylococcus aureus bloodstream infections and 20,000 associated deaths occurring annually in the United States alone^1^. MRSA infection is one of the leading causes of hospital-acquired infections and is commonly associated with significant morbidity, mortality, prolonged hospital stays, and substantial cost burden^2^. The emergence and spread of MRSA strains, particularly those exhibiting resistance to multiple antibiotics, have complicated treatment strategies and necessitated the urgent development of alternative antimicrobial approaches^3,4^. Recent epidemiological data indicate that while hospital-onset MRSA infections have decreased, community-acquired MRSA cases continue to pose significant public health concerns, highlighting the persistent threat of this pathogen^5^. The formation of biofilms by MRSA further exacerbates treatment challenges, as biofilm-associated bacteria demonstrate 10–1000 times greater resistance to antimicrobial agents compared to planktonic cells^6^.

Essential oils (EOs) are complex mixtures of volatile secondary metabolites derived from aromatic plants, containing bioactive compounds with demonstrated antimicrobial, antioxidant, and anti-inflammatory properties^7,8^. Oregano essential oil (*Origanum vulgare* L.), belonging to the Lamiaceae family, has emerged as a particularly promising natural antimicrobial agent due to its high content of phenolic compounds, primarily carvacrol (15.8–85.70%) and thymol (1.65–52.83%)^9,10^. These monoterpene phenols exhibit potent antibacterial activity against both Gram-positive and Gram-negative bacteria through multiple mechanisms, including membrane disruption, enzyme inhibition, and interference with cellular metabolism^11,12^. Recent studies have demonstrated that oregano essential oil exhibits significant antimicrobial efficacy against MRSA strains, with minimum inhibitory concentrations ranging from 0.08% to 0.64% (v/v)^13,14^. Among these bioactive compounds, thymol has garnered particular attention as a single-component antimicrobial agent due to its well-characterized structure-activity relationships and reproducible therapeutic effects compared to complex essential oil mixtures. The isolation and utilization of pure thymol enable precise dosage control and eliminates the variability inherent in essential oil compositions, making it an ideal candidate for pharmaceutical nanoemulsion development.

Despite their promising antimicrobial properties, the practical application of essential oils is significantly limited by several inherent challenges. These include poor water solubility, high volatility leading to rapid evaporation and loss of bioactive compounds, susceptibility to environmental degradation, and low bioavailability in aqueous systems^15,16^. The hydrophobic nature of essential oils results in uneven distribution in aqueous media, while their volatile characteristics lead to uncontrolled release and reduced therapeutic efficacy^17^. Additionally, the strong organoleptic properties and potential for skin irritation at effective concentrations create formulation challenges for pharmaceutical applications^18^. These limitations necessitate the development of advanced delivery systems that can protect essential oils from degradation while enhancing their stability, bioavailability, and controlled release characteristics^19^.

Nanoemulsions represent an advanced form of novel drug delivery systems designed to enhance the solubility and bioavailability of poorly water-soluble drugs^20^. Their nanoscale droplet size provides a large surface area, facilitating improved drug absorption and controlled release^21^. Additionally, nanoemulsions offer physical stability, ease of preparation, and compatibility with both hydrophilic and lipophilic drugs^22^. Overall, they hold significant potential for targeted and efficient therapeutic delivery in pharmaceutical and biomedical applications^23^.

To address these challenges, this study aimed to develop thymol-loaded nanoemulsions as an innovative delivery system for enhanced antimicrobial activity against MRSA. This comprehensive approach aims to establish nanoemulsion technology as a viable strategy for enhancing the therapeutic potential of thymol in combating MRSA infections.

## Materials and methods

### Materials

Dimethyl sulfoxide (DMSO) was purchased from Shanghai Aladdin Biochemical Technology Co., Ltd. (Shanghai, China). Propidium iodide (PI, 10 µg/mL), 6-dodecanoyl-N, N-dimethyl-2-naphthylamine (Laurdan), 3,3’-dipropylthiadicarbocyanine iodide [DiSC₃(5)], and 2’,7’-dichlorodihydrofluorescein diacetate (DCFH-DA) were obtained from Shanghai Aladdin Biochemical Technology Co., Ltd. (Shanghai, China). Oxacillin and formaldehyde were purchased from Beijing Solarbio Science & Technology Co., Ltd. (Beijing, China). Thymol was obtained from Shanghai Macklin Biochemical Technology Co., Ltd. (Shanghai, China). Tween 80 (polyoxyethylene sorbitan monooleate) and diethylene glycol monoethyl ether (DEGME) were used as surfactant and co-surfactant, respectively. All chemicals were of analytical grade and used without further purification. Distilled water was used throughout all experiments.

### Bacterial strains and culture conditions

All bacterial strains used in this study were obtained from Beina Biotechnology Co., Ltd. (Beijing, China). The strains were preserved at −80℃ and subcultured in Mueller-Hinton broth (MHB) for revival. Prior to experiments, frozen stocks were thawed and cultured in MHB at 35–37℃with continuous shaking at 180 rpm for 16 h to achieve logarithmic growth phase. Fresh bacterial suspensions were prepared by adjusting the optical density at 600 nm (OD_600_) to 0.5 McFarland standard, corresponding to approximately 1.5 × 10^8^ CFU/mL.

### Optimization of the preparation process of thymol nanoemulsion (TNE)

#### Single-factor optimization

Single-factor experiments were conducted to investigate the individual effects of key formulation parameters on nanoemulsion characteristics and to determine the appropriate ranges for subsequent response surface methodology (RSM). Three critical factors were evaluated: oil phase percentage (X_1_), surfactant percentage (X_2_), and Smix ratio (X_3_, Tween80/DEGME ratio). Each factor was varied while keeping other parameters constant.

For oil phase percentage, the range of 2–10% (w/w) was investigated at 1% intervals. Surfactant percentage was evaluated from 10 to 25% (w/w) at 2.5% intervals, while Smix ratio was tested at 0.5:1, 1:1, 1.5:1, 2:1, 2.5:1, 3:1, and 4:1. The aqueous phase percentage was automatically determined to maintain the total formulation at 100%.

Based on single-factor results, the optimal ranges for RSM were determined as: oil phase 4–6%, surfactant percentage 15–20%, and Smix ratio 2:1–3:1. These ranges were selected based on achieving minimum particle size, low polydispersity index (PDI), and moderate zeta potential values.

#### Particle size and zeta potential analysis

The TNE was appropriately diluted with distilled water prior to measurement. Particle size, polydispersity index (PDI), and zeta potential were determined using a Zetasizer Nano ZS90 (Malvern Instruments Ltd., UK) at room temperature. Each sample was analyzed in triplicate to ensure reproducibility^24^.

#### Central composite response surface design

A three-factor, three-level Central Composite Design (CCD) was employed to optimize the nanoemulsion formulation and investigate factor interactions^25^. The independent variables and their levels are presented in Table 1. Twenty experimental runs were generated, including 8 factorial points, 6 star points, and 6 center points for error estimation.

**Table 1:** Independent variables and their levels used in Central Composite design.

Factors	Symbol	−1 Level	0 Level	+ 1 Level
Oil phase percentage	X₁	4%	5%	6%
Surfactant percentage	X₂	15%	17.5%	20%
Smix ratio	X₃	2	2.5	3

#### Multi-response optimization

A desirability function approach was employed to simultaneously optimize multiple responses. Individual desirability functions were calculated for each response variable using the following equations:

For “smaller-the-better” characteristics (Z-Average and PDI):

$$d_{1} = ({\mathrm{Z-Average}}_{\max}- {\mathrm{Z-Average}})/({\mathrm{Z-Average}}_{\max} - {\mathrm{Z-Average}}_{\min})$$

$$d_{2} = ({\rm PDI}_{\max} - {\rm PDI})/({\rm PDI}_{\max} - {\rm PDI}\_\min)$$

For zeta potential (moderate absolute value preferred):

$$d_{3} = (|{\text{Zeta potential}}|_{\max} - |{\text{Zeta potential}}|)/(|{\text{Zeta potential}}|_{\max} - |{\text{Zeta potential}}|_{\min}).$$

The overall desirability (OD) was calculated as the geometric mean of individual desirabilities: OD = (d_1_ × d_2_ × d_3_) ^ (1/3).

where d_1_, d_2_, and d_3_ represent the individual desirability functions for Z-Average, PDI, and zeta potential, respectively.

#### Statistical analysis

Response surface models were fitted using second-order polynomial equations. Analysis of variance (ANOVA) was performed to evaluate model significance and adequacy. Model validation was assessed using coefficient of determination (R^2^), adjusted R^2^, and lack-of-fit tests. All statistical analyses were conducted using Design-Expert software (version 13.0, Stat-Ease Inc., Minneapolis, MN, USA) at a significance level of *p* < 0.05.

#### Verification experiments

The optimized formulation predicted by the desirability function was prepared in triplicate to validate the model accuracy. The experimental values were compared with predicted values to calculate the relative error and confirm the reliability of the optimization approach.

### Nanoemulsion type determination

The type of prepared nanoemulsion was determined using the classical dual-dye diffusion method. Equal volumes of freshly prepared nanoemulsion samples were placed in separate glass vials, followed by addition of 2 drops of 0.1% methylene blue aqueous solution and 0.1% oil red O ethanol solution, respectively. After gentle mixing, the samples were allowed to stand for 10 min, and the diffusion behavior of each dye was observed. Water-soluble dyes disperse rapidly and uniformly in oil-in-water (O/W) nanoemulsions, while oil-soluble dyes show preferential distribution in the dispersed phase, confirming the nanoemulsion type.

### Antibacterial activity of TNE

The minimum inhibitory concentrations (MIC) of TNE and free thymol against various bacterial strains were determined according to the Clinical and Laboratory Standards Institute (CLSI) guidelines. Blank nanoemulsion (without thymol) and oxacillin sodium served as negative and positive controls, respectively.

### Membrane damage assessment of TNE

Strains were retrieved from − 80℃ stock cultures and activated on Mueller-Hinton agar (MHA) plates at 37℃ for 24 h. Single colonies were inoculated into 5 mL of MHB and cultured overnight at 37℃ with shaking at 150 rpm. Subsequently, 50 *µ*L of the activated culture was transferred to 5 mL of fresh MHB and incubated at 37℃ with shaking at 150 rpm until reaching logarithmic growth phase (approximately 3–4 h, OD_600_ = 0.4–0.6).

The bacterial suspension was aliquoted into sterile centrifuge tubes and treated with different concentrations (0, 0.5×MIC, 1×MIC, 2×MIC) of thymol nanoemulsion. Samples were incubated at 37℃ with shaking at 150 rpm for 2 h to allow sufficient interaction between the nanoemulsion and bacterial cells. After treatment, cells were harvested by centrifugation at 5,000 rpm for 5 min, washed three times with sterile phosphate-buffered saline (PBS, pH 7.4) to remove residual medium and unbound essential oil. Finally, the bacterial pellet was resuspended in PBS and adjusted to OD_600_ = 0.5 (approximately 1.5 × 10^8^ CFU/mL) to prepare standardized bacterial suspensions for subsequent membrane function assays. Sufficient volumes were prepared for each treatment group to ensure all membrane function tests used samples from the same batch, minimizing experimental variation.

#### Cell surface hydrophobicity determination

Cell surface hydrophobicity was determined using the microbial adhesion to hydrocarbons (MATH) method, which evaluates cell surface hydrophobicity based on bacterial partitioning behavior between aqueous and hydrocarbon phases^26^. Three milliliters of standardized bacterial suspension were transferred to clean, sterile glass tubes, and the initial OD_600_ value (OD_0_) was recorded. Subsequently, 0.3 mL of chromatography-grade n-hexadecane was added to each tube (hydrocarbon to sample volume ratio of 1:10) and vortexed vigorously for 2 min at 2,800 rpm to ensure thorough contact. The mixture was then allowed to stand for 15 min at room temperature for complete phase separation. The lower aqueous phase was carefully aspirated without disturbing the interface, and the OD_600_ value (OD_1_) was measured again. The decrease in aqueous phase OD_600_ reflected the extent of bacterial migration to the organic phase, indirectly indicating enhanced cell surface hydrophobicity. Each concentration was tested in triplicate, and experiments were repeated three times.

Bacterial hydrophobicity was expressed as adhesion rate, calculated using the formula: Adhesion rate (%) = (OD_0_ – OD_1_)/OD_0_ × 100%.

where OD₀ represents the OD₆₀₀ value before n-hexadecane addition, and OD_1_ represents the OD_600_ value of the aqueous phase after hydrocarbon extraction.

#### Cell membrane integrity analysis

The effect of nanoemulsion on MRSA cell membrane integrity was evaluated using propidium iodide (PI), a fluorescent dye that cannot penetrate intact cell membranes. PI enters only membrane-damaged cells and binds to DNA, emitting red fluorescence proportional to the degree of membrane damage.

Two hundred microliters of standardized bacterial suspension from each treatment group were placed in 96-well black-bottom clear microtiter plates. Ten microliters of PI solution (10 µg/mL in PBS) were added to each well to achieve a final concentration of 0.5 µg/mL. After mixing, samples were incubated at 37℃ in the dark for 30 min to allow sufficient PI binding to DNA in membrane-compromised cells. Following incubation, samples were centrifuged at 3,000 rpm for 5 min to remove unbound PI, washed once with PBS, and finally resuspended in 200 µL of PBS.

Fluorescence intensity was measured using a microplate reader at excitation wavelength 535 nm and emission wavelength 615 nm. To correct for potential differences in cell numbers between treatment groups, OD_600_ values were simultaneously measured, and fluorescence intensity per unit OD_600_ (FI/OD_600_) was calculated as the final result. Samples treated with 70% ethanol for 5 min served as positive controls (complete membrane disruption), while untreated bacterial suspensions served as negative controls (baseline membrane permeability). Each concentration was tested in six replicates, and experiments were repeated three times.

Membrane damage was expressed as relative fluorescence intensity: Relative fluorescence intensity (%) = (Treatment FI/OD_600_ - Negative control FI/OD_600_)/(Positive control FI/OD_600_ - Negative control FI/OD_600_ × 100%.

#### Effect on MRSA membrane fluidity

The impact of nanoemulsion treatment on MRSA membrane fluidity was assessed using the environment-sensitive fluorescent probe 6-dodecanoyl-N, N-dimethyl-2-naphthylamine (Laurdan). Laurdan exhibits unique fluorescence characteristics in lipid environments, with its emission spectrum changing according to surrounding environmental polarity, making it suitable for monitoring membrane lipid phase state and fluidity changes.

Bacterial suspensions from each treatment group were adjusted to OD_600_ = 0.4, and 200 *µ*L were placed in 96-well black-bottom clear microtiter plates. Two microliters of Laurdan stock solution (10 mM in DMSO) were added to achieve a final concentration of 10 *µ*M. After mixing, samples were incubated at 37℃ in the dark for 30 min to allow complete Laurdan insertion into cell membranes. Following incubation, samples were centrifuged at 3,000 rpm for 5 min to remove unbound Laurdan, washed once with PBS, and finally resuspended in 200 *µ*L of PBS.

Using a fluorescence microplate reader with excitation wavelength set at 340 nm, emission fluorescence intensities were measured at 440 nm and 490 nm (I_440_ and I_490_, respectively). Membrane fluidity was calculated using the generalized polarization (GP) formula:

$${\rm GP} = (I_{440}-I_{490})/(I_{440}+I_{490}).$$

GP values range from − 1 to + 1, with increased values indicating decreased environmental polarity and increased membrane order (decreased membrane fluidity), while decreased values indicate increased membrane fluidity. Each concentration was tested in six replicates, and experiments were repeated three times.

#### Effect on MRSA membrane potential

The effect of nanoemulsion on MRSA membrane potential was detected using the lipophilic cationic fluorescent dye 3,3’-dipropylthiadicarbocyanine iodide [DiSC_3_(5)]. Under normal conditions, negatively charged bacterial cell membranes maintain transmembrane potential, allowing DiSC_3_(5) to distribute into the membrane and causing fluorescence self-quenching. When membrane potential is lost, the dye is released extracellularly, resulting in increased fluorescence intensity.

Bacterial suspensions from each treatment group were adjusted to OD_600_ = 0.1, and 1 mL was placed in sterile fluorescence cuvettes. Two microliters of DiSC_3_(5) stock solution (1 mM in DMSO) were added to achieve a final concentration of 2 *µ*M. Samples were incubated at room temperature in the dark for 20 min to allow probe equilibration with cells. Subsequently, fluorescence intensity changes were continuously monitored for 60 s (recorded every 10 s) using a fluorescence spectrophotometer at excitation wavelength 622 nm and emission wavelength 670 nm. Average values were calculated as final results.

Relative fluorescence intensity increase (%) = [(Treatment _FI_ - Control _FI_)/Control _FI_] × 100%.

#### Lactate dehydrogenase (LDH) content determination

LDH is a stable cytoplasmic enzyme whose release serves as an important indicator of cell membrane damage. After treating bacterial suspensions for 2 h according to the method described above, supernatants were collected by centrifugation at 5,000 rpm for 10 min. LDH content was determined using a Beyotime LDH Detection Kit following the manufacturer’s standard operating procedures.

The procedure involved adding 120 µL of supernatant and 60 µL of working solution (containing NAD^+^, lactate, tetrazolium, and diaphorase) to 96-well plates, followed by incubation at 37 ℃ in the dark for 30 min. LDH catalyzes lactate oxidation to pyruvate while reducing NAD⁺ to NADH, which subsequently reacts with tetrazolium to produce colored formazan proportional to LDH activity. Absorbance was measured at 490 nm using a microplate reader, with 630 nm serving as the reference wavelength for background correction. Each concentration was tested in six replicates, and experiments were repeated three times.

### Statistical analysis

Data analysis was performed using GraphPad Prism software (GraphPad Software Inc., San Diego, CA, USA). Results are expressed as mean ± standard deviation (SD). Statistical analysis was conducted using one-way analysis of variance (ANOVA) followed by Tukey’s multiple comparison test. Differences were considered statistically significant at *p* < 0.05. All experiments were performed in triplicate with at least three independent biological replicates to ensure reproducibility and statistical validity.

## Result

### Single-factor optimization

**Fig. 1:**
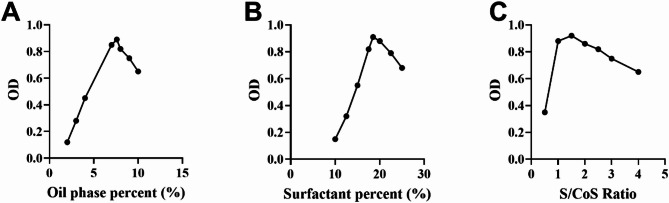
Effects of single factors on nanoemulsion characteristics: (**A**) oil phase percentage, (**B**) surfactant percentage, and (**C**) Smix ratio on particle size.

Single-factor experiments were conducted to evaluate the individual effects of formulation parameters on nanoemulsion characteristics and establish appropriate ranges for response surface methodology. The effects of oil phase percentage, surfactant percentage, and Smix ratio on particle size, polydispersity index (PDI), and zeta potential are presented in Fig. 1.

As shown in Fig. 1A, oil phase percentage significantly influenced the overall desirability (OD). OD increased initially with increasing oil phase percentage from 2% to 8%, reaching maximum values around 8%, then gradually decreased beyond 8%. This trend can be attributed to the balance between droplet formation and coalescence. Lower oil concentrations may result in insufficient droplet formation, while excessive oil content can lead to increased viscosity and larger droplets, ultimately affecting the comprehensive performance indicators.

Figure 1B demonstrates the effect of surfactant percentage on nanoemulsion overall desirability. Increasing surfactant concentration from 5% to 20% resulted in significant OD improvement, indicating enhanced emulsification efficiency and better comprehensive properties. However, further increases beyond 20% showed decreased OD values, suggesting that excessive surfactant concentration may negatively impact certain nanoemulsion characteristics.

The influence of Smix ratio is presented in Fig. 1C. The optimal Smix ratio was found to be around 2:1, where OD reached maximum values around 0.92. At lower ratios (0.5:1–1:1), the OD was relatively lower, while higher ratios (> 3:1) also resulted in decreased OD values. Based on these results, the factor ranges for response surface design were established as: oil phase 6–10%, surfactant 15–25%, and Smix ratio 1:1–3:1.

### Response surface methodology

#### Experimental results

The Central Composite Design (CCD) encompassed 20 experimental runs with three independent variables: oil phase percentage (4–6%), surfactant percentage (15–20%), and S/mix ratio (2–3) (Table 2). The response variables demonstrated considerable variation across formulations (Table 3). The overall desirability (OD) values ranged from 0 to 0.851695, with run 15 achieving the highest score (0.851695), followed by run 18 (0.844823) and run 16 (0.829359). Runs 2 and 13 showed the poorest performance with OD values of 0, indicating unfavorable formulation conditions. The star points (runs 9–14) explored the extended design space beyond the factorial region, with run 9 showing particularly good performance (OD = 0.802996). The center point runs (15–20) demonstrated good reproducibility with OD values ranging from 0.778249 to 0.851695 (mean = 0.823018, RSD = 3.5%), confirming the stability of the experimental design and the absence of significant time-related drift during experimentation. This variation in OD values across different formulations demonstrates the significant impact of formulation parameters on the overall nanoemulsion quality and the effectiveness of the CCD in identifying optimal conditions within the explored design space.

**Table 2:** Central composite experimental design.

Std	oil phase percent (%)	surfactant percent (%)	S/CoS Ratio
1	4	15	2
2	6	15	2
3	4	20	2
4	6	20	2
5	4	15	3
6	6	15	3
7	4	20	3
8	6	20	3
9	3.3	17.5	2.5
10	6.7	17.5	2.5
11	5	13.3	2.5
12	5	21.7	2.5
13	5	17.5	1.6
14	5	17.5	3.3
15	5	17.5	2.5
16	5	17.5	2.5
17	5	17.5	2.5
18	5	17.5	2.5
19	5	17.5	2.5
20	5	17.5	2.5

**Table 3:** Central composite design experimental design matrix and response values for thymol nanoemulsion optimization.

Std	oil phase percent (%)	surfactant percent (%)	Smix Ratio	OD
1	4	15	2	0.217113
2	6	15	2	0
3	4	20	2	0.616712
4	6	20	2	0.280164
5	4	15	3	0.605031
6	6	15	3	0.463506
7	4	20	3	0.676993
8	6	20	3	0.44452
9	3.3	17.5	2.5	0.802996
10	6.7	17.5	2.5	0.506496
11	5	13.3	2.5	0.254566
12	5	21.7	2.5	0.513749
13	5	17.5	1.6	0
14	5	17.5	3.3	0.523151
15	5	17.5	2.5	0.851695
16	5	17.5	2.5	0.829359
17	5	17.5	2.5	0.814721
18	5	17.5	2.5	0.844823
19	5	17.5	2.5	0.778249
20	5	17.5	2.5	0.81843

#### Model development and statistical analysis

Second-order polynomial regression models were developed to correlate formulation variables with responses. ANOVA results demonstrated excellent model significance (F = 191.17, *p* < 0.0001) with high predictive capability (R^2^ = 0.9942) (Table 4). The lack of fit was not significant (*p* = 0.3560), confirming model adequacy. All linear and quadratic terms showed high significance, with X₂X₃ interaction being the most significant interaction term,

**Table 4:** ANOVA of regression model for overall desirability (OD).

Source	Sum of Squares	df	Mean Square	F-value	*p*-value	
Model	1.43	9	0.1585	191.17	< 0.0001	Significant
X_1_	0.1490	1	0.1490	179.64	< 0.0001	
X_2_	0.1000	1	0.1000	120.59	< 0.0001	
X_3_	0.2801	1	0.2801	337.80	< 0.0001	
X_1_ × _2_	0.0055	1	0.0055	6.67	0.0273	
X_1_ × _3_	0.0040	1	0.0040	4.87	0.0519	
X_2_ × _3_	0.0491	1	0.0491	59.22	< 0.0001	
X_1_²	0.0498	1	0.0498	60.05	< 0.0001	
X_2_²	0.3438	1	0.3438	414.57	< 0.0001	
X_3_²	0.5638	1	0.5638	679.88	< 0.0001	
Residual	0.0083	10	0.0008			
Lack of Fit	0.0049	5	0.0010	1.42	0.3560	Not significant
Pure Error	0.0034	5	0.0007			
Cor Total	1.71	19				

#### Response surface analysis

**Fig. 2:**
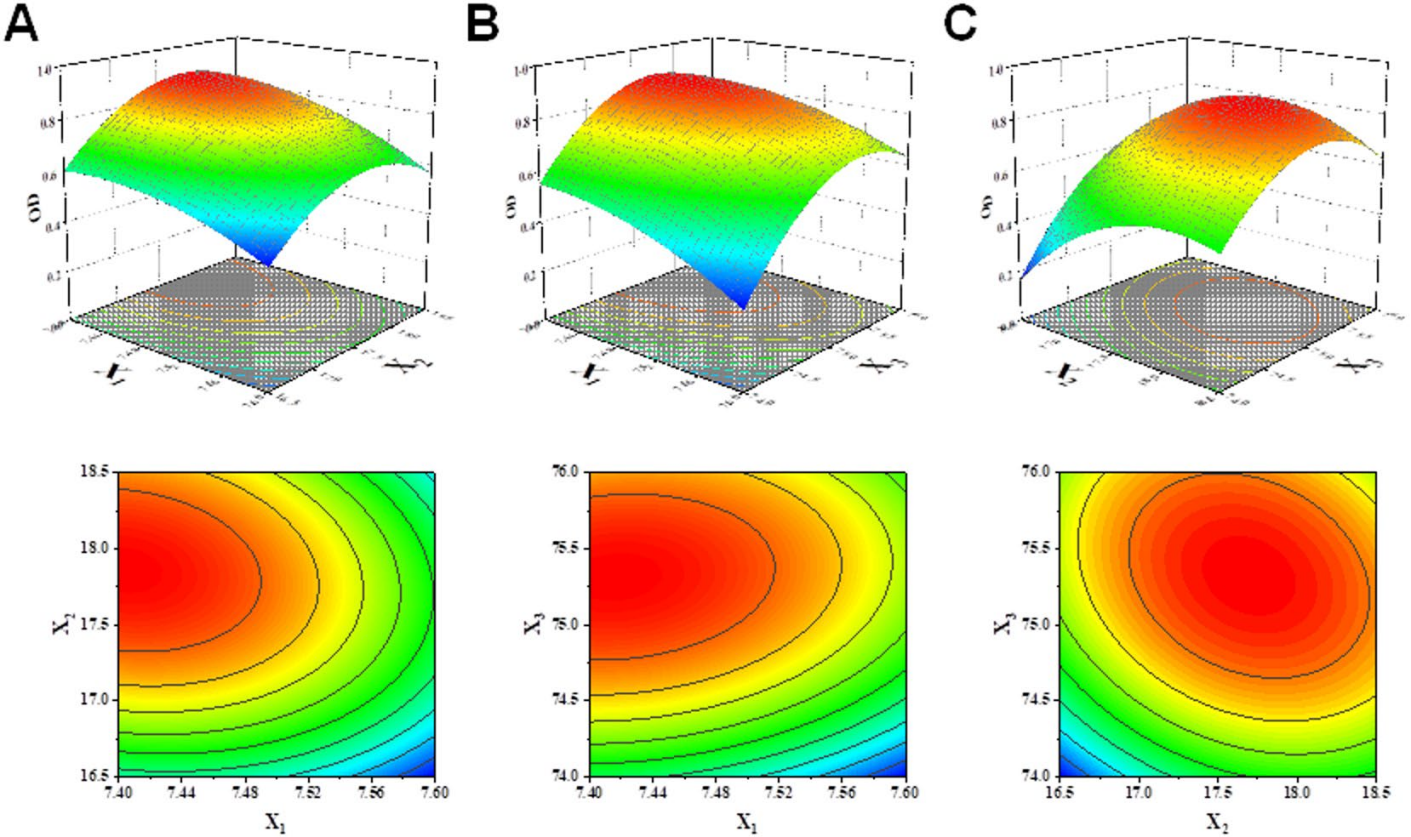
Response surface plots showing the effects of formulation variables on (**A**) particle size, (**B**) PDI, and (**C**) zeta potential.

Response surface plots revealed the effects of formulation variables on nanoemulsion properties (Fig. 2). Particle size was primarily influenced by surfactant percentage, with optimal reduction achieved at higher concentrations combined with moderate oil phase levels. PDI showed similar trends, with the most uniform distributions obtained at higher surfactant concentrations. Zeta potential demonstrated complex interaction patterns, with moderate values (−3 to −5 mV) considered optimal.

#### Optimization and validation

Multi-response optimization using desirability function identified the optimal formulation: oil phase 4.6%, surfactant 18.5%, and Smix ratio 2.5:1, achieving the highest overall desirability (OD = 0.925). As shown in Table 5, experimental validation showed excellent reproducibility with actual OD values of 0.924 ± 0.002 (RSD = 0.16%), confirming close agreement with predicted values (relative error = 0.11%) and demonstrating the reliability of the optimization approach and suitability of the optimized nanoemulsion for pharmaceutical applications.

**Table 5:** Optimal prescription verification experiment results.

Rank	OD_Actual_	OD_Forecast_	RSD%
1	0.923	0.925	0.16
2	0.926		
3	0.924		

### Nanoemulsion characterization

The dual-dye diffusion test confirmed the formation of an oil-in-water (O/W) nanoemulsion, as methylene blue dispersed rapidly throughout the system while oil red O remained in the oil droplets. The optimized formulation yielded nanoemulsions with particle size of 25.51 ± 0.15 nm, PDI of 0.114 ± 0.008, and zeta potential of −2.73 ± 0.12 mV, demonstrating excellent agreement with response surface predictions and confirming successful nanoemulsion formation.

### Antibacterial activity of TNE

TNE demonstrated consistent antimicrobial activity with MIC values of 15 mg/mL (corresponding to 690 µg/mL thymol) against all tested strains, including MRSA ATCC 43,300, S. aureus ATCC 25,923, and E. coli strains ATCC 43,895 and ATCC 25,922 (Table 6). The effective thymol concentration in TNE (690 µg/mL) was lower than that of free thymol (800 µg/mL), indicating enhanced antimicrobial efficacy of the nanoemulsion formulation. Blank nanoemulsion showed no activity, while oxacillin sodium exhibited MIC values ranging from 1 µg/mL (S. aureus) to 32 µg/mL (MRSA), reflecting typical β-lactam resistance.

**Table 6:** MIC of TNE and controls against bacterial strains.

Strains	TNE (mg/mL)	Thymol content in TNE (µg/mL)	NE	Thymol (µg/mL)	Oxacillin Sodium (µg/mL)
MRSA ATCC 43,300	15	690	/	800	32
*S. aureus* ATCC25923	15	690	/	800	1
*E.coli* ATCC 43,895	15	690	/	800	8
*E.coli* ATCC 25,922	15	690		800	8

### Membrane damage mechanisms of TNE

**Fig. 3:**
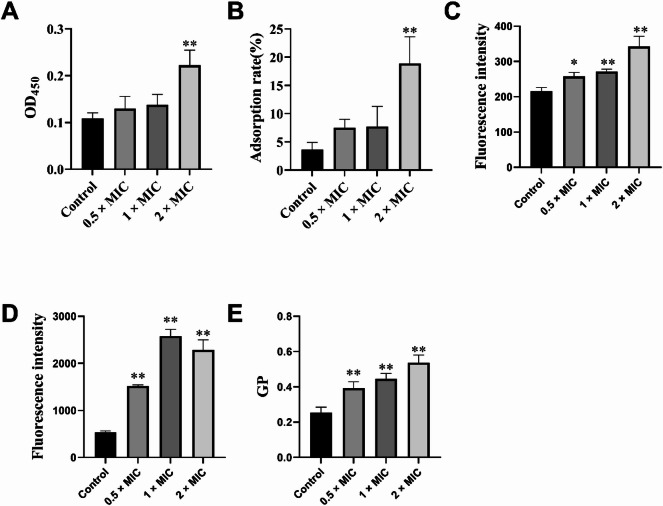
Membrane damage mechanisms of thymol nanoemulsion against MRSA.

The antimicrobial mechanisms of the optimized nanoemulsion against MRSA were evaluated through multiple cellular integrity assays, as shown in Fig. 3. The nanoemulsion demonstrated dose-dependent effects on bacterial membrane integrity. LDH leakage assays (Fig. 3A) revealed significant membrane damage, with substantial increases in cytoplasmic leakage at higher concentrations compared to the control group (*p* < 0.01). The MATH assay (Fig. 3B) showed a marked dose-dependent increase in bacterial cell surface hydrophobicity, with the highest concentration (2×MIC) demonstrating significantly enhanced adsorption rates compared to untreated controls (*p* < 0.01), suggesting alterations in membrane composition and structure.

Membrane permeability studies using propidium iodide staining (Fig. 3C) demonstrated concentration-dependent increases in fluorescence intensity, with all treatment groups showing significantly higher membrane permeability than controls, particularly at 2×MIC (*p* < 0.01). The membrane potential analysis using DiSC_3_(5) fluorescent probe (Fig. 3D) revealed progressive membrane depolarization effects, with treatment groups exhibiting significantly elevated fluorescence compared to controls, indicating severe disruption of the electrochemical gradient across the bacterial membrane (*p* < 0.01).

Membrane fluidity measurements using Laurdan generalized polarization values (Fig. 3E) showed dose-dependent increases in membrane rigidity, with all nanoemulsion concentrations significantly increasing GP values compared to untreated controls (*p* < 0.01). These comprehensive membrane integrity assessments collectively demonstrate that the nanoemulsion exerts its antimicrobial effects primarily through multiple membrane-targeting mechanisms, including membrane permeabilization, depolarization, hydrophobicity alteration, and rigidity modulation, ultimately leading to bacterial cell death.

## Discussion

The development of thymol-loaded nanoemulsions represents a significant advancement in addressing the formulation challenges associated with essential oil-based antimicrobial agents. Recent studies have demonstrated that essential oil nanoemulsions with particle sizes below 50 nm exhibit enhanced antimicrobial activity due to increased surface area and improved membrane interaction^27,28^. The achieved particle size of 25.51 ± 0.15 nm with excellent monodispersity (PDI = 0.114 ± 0.008) demonstrates successful nanoemulsion formation. The low PDI value indicates narrow size distribution, which is crucial for consistent antimicrobial performance and product stability^29^.

The pseudo-ternary phase diagram approach employed in our optimization proved highly effective, consistent with established nanoemulsion development methodologies^30^. The optimal formulation parameters 4.6% oil phase, 18.5% surfactant, and 2.5:1 surfactant-to-cosurfactant ratio align with theoretical predictions from HLB considerations for oil-in-water systems^31^. Compared to the recently reported *Schizonepeta annua* essential oil nanoemulsion system^32^, our thymol-based formulation offers advantages in reproducibility and standardization by using a purified compound rather than crude essential oil.

The comprehensive mechanistic evaluation revealed that TNE exerts antimicrobial effects through multiple pathways targeting bacterial membrane integrity, consistent with established mechanisms of essential oil action^7,11^. Our findings of dose-dependent membrane permeabilization, depolarization, and altered surface hydrophobicity align closely with previous reports on phenolic monoterpenes^12,33^. Particularly noteworthy is our observation of increased membrane rigidity concurrent with permeabilization. While seemingly contradictory, this likely represents thymol-induced phase transition that disrupts the optimal fluid-mosaic structure required for membrane protein function, similar to effects reported for other phenolic compounds^34^. This multi-target nature is particularly significant in the context of antibiotic resistance, as it reduces the likelihood of resistance development compared to conventional single-target antibiotics^35^.

The consistent MIC values of 15 mg/mL against MRSA and other bacterial strains demonstrate successful translation into a pharmaceutically viable system. The thymol content of690 µg/mL in our nanoemulsion formulation achieves antimicrobial efficacy comparable to previous reports on essential oil-based systems, while the nanoemulsion structure addresses inherent limitations of free essential oils including poor water solubility, high volatility, and rapid degradation^32^. Cytotoxicity studies with HaCaT keratinocytes revealed that SEO-NE maintained good biocompatibility at antimicrobial effective concentrations, with a favorable therapeutic window where antimicrobial efficacy (3.9 mg/mL) was achieved well below cytotoxic concentrations (≥ 64 mg/mL). This improved safety profile can be attributed to the nanoemulsion structure modulating interactions between bioactive components and cellular membranes^36^.

The clinical relevance of this research is substantial given the persistent challenges with MRSA infections. MRSA remains one of the most important causes of hospital-acquired infections, with approximately 120,000 bloodstream infections and 20,000 associated deaths occurring annually in the United States^37^. The formation of biofilms by MRSA further complicates treatment, as biofilm-associated bacteria demonstrate 10–1000 times greater resistance to antimicrobial agents compared to planktonic cells^38^. Although our current study focused on planktonic MRSA, the multi-target membrane disruption mechanism we documented suggests potential efficacy against biofilm-associated bacteria, warranting future investigation.

Future research should focus on several key areas to advance this technology toward clinical application. First, comprehensive in vivo efficacy studies in appropriate animal models are essential to establish therapeutic potential and identify optimal dosing regimens. Second, detailed safety and toxicology assessments are needed, including chronic toxicity studies and assessment of potential impacts on the normal microbiome. Third, investigation of potential synergistic applications with existing antimicrobial therapies could enhance efficacy while reducing required concentrations^39^. Finally, evaluation of anti-biofilm properties would address a critical clinical need, as biofilm-associated infections are notoriously difficult to treat.

In conclusion, this study successfully developed and characterized optimized thymol-loaded nanoemulsions with potent antimicrobial activity against MRSA. The comprehensive mechanistic investigation revealed multi-target membrane disruption as the primary mode of action, suggesting lower resistance potential compared to conventional antibiotics. These findings establish a strong foundation for future translational research, representing a promising platform for combating multidrug-resistant infections. However, several limitations should be acknowledged for future investigation, including the need for biofilm efficacy evaluation, in vivo pharmacological studies, extended stability assessment following ICH guidelines, and comprehensive cytotoxicity evaluation to support clinical translation.

## Data Availability

All data generated or analysed during this study are included in this published article.
